# Improved definition of the mouse transcriptome via targeted RNA sequencing

**DOI:** 10.1101/gr.199760.115

**Published:** 2016-05

**Authors:** Giovanni Bussotti, Tommaso Leonardi, Michael B. Clark, Tim R. Mercer, Joanna Crawford, Lorenzo Malquori, Cedric Notredame, Marcel E. Dinger, John S. Mattick, Anton J. Enright

**Affiliations:** 1 EMBL, European Bioinformatics Institute, Cambridge, CB10 1SD, United Kingdom;; 2 Garvan Institute of Medical Research, Sydney, New South Wales 2010, Australia;; 3 MRC Functional Genomics Unit, Department of Physiology, Anatomy, and Genetics, University of Oxford, Oxford OX1 3PT, United Kingdom;; 4 St Vincent's Clinical School, UNSW Australia, Sydney, New South Wales 2052, Australia;; 5 Institute for Molecular Bioscience, The University of Queensland, Brisbane, Queensland 4072, Australia;; 6 Comparative Bioinformatics, Bioinformatics and Genomics Program, Centre for Genomic Regulation (CRG), 08003 Barcelona, Spain

## Abstract

Targeted RNA sequencing (CaptureSeq) uses oligonucleotide probes to capture RNAs for sequencing, providing enriched read coverage, accurate measurement of gene expression, and quantitative expression data. We applied CaptureSeq to refine transcript annotations in the current murine GRCm38 assembly. More than 23,000 regions corresponding to putative or annotated long noncoding RNAs (lncRNAs) and 154,281 known splicing junction sites were selected for targeted sequencing across five mouse tissues and three brain subregions. The results illustrate that the mouse transcriptome is considerably more complex than previously thought. We assemble more complete transcript isoforms than GENCODE, expand transcript boundaries, and connect interspersed islands of mapped reads. We describe a novel filtering pipeline that identifies previously unannotated but high-quality transcript isoforms. In this set, 911 GENCODE neighboring genes are condensed into 400 expanded gene models. Additionally, 594 GENCODE lncRNAs acquire an open reading frame (ORF) when their structure is extended with CaptureSeq. Finally, we validate our observations using current FANTOM and Mouse ENCODE resources.

Advances in sequencing technology have permitted rapid high-throughput sequencing of cDNA and the discovery of novel genes and transcript isoforms. This has also fostered the rapid accumulation of annotated long noncoding RNAs (lncRNAs) (Bussotti et al. 2013) and further recognition that the vast majority of genes express alternative isoforms (Katz et al. 2015). Current estimates of human lncRNA loci range from 58,648 from a large compendium of RNA-seq data sets (Iyer et al. 2015) to 15,900 lncRNA loci (27,670 transcripts) in the more conservative GENCODE catalog (Harrow et al. 2012). The FANTOM3 Consortium identified 34,040 mouse cDNAs lacking coding capability (The FANTOM Consortium et al. 2005; Maeda et al. 2006), with only 6951 lncRNA loci (9962 transcripts) currently annotated in GENCODE (M4). This number discrepancy suggests a pressing need to increase and improve mouse gene annotations to make them comparable to those that exist for human.

LncRNAs are commonly defined as transcripts longer than 200 bases lacking the potential to be translated into proteins (Derrien et al. 2012). They exhibit a wide range of lengths. For example, *XIST* has a 19-kb isoform in human (Pontier and Gribnau 2011), whereas *NRON* (Willingham et al. 2005) is just over 600 nt. They characteristically exhibit low expression (mostly 0.1–0.001 fragments per kilobase of transcript per million mapped reads [FPKM] in human), considerably below the protein-coding expression range (0.1–10 FPKMs) (Derrien et al. 2012). Recently, there has been increasing evidence that lncRNAs serve crucial biological roles (Mattick 2009). Examples include organism development (Amaral et al. 2009; Mattick 2011), imprinting (Zhang et al. 2014), epigenetic control (Nakagawa and Kageyama 2014), X inactivation (Froberg et al. 2013), and cancer etiology (Yang et al. 2014).

The discovery of functional but rare RNAs is limited by the sensitivity of sequencing methods to low-abundance transcripts and may be improved by depletion of ribosomal RNA or protein-coding transcripts. Previous work (Jiang et al. 2011) showed that 43% of sequencing reads aligned to the 1.5% most common transcripts, whereas only 1% of reads mapped to the 44% least abundant transcripts. This low depth of coverage inhibits current ab initio or de novo assemblers from identifying accurate transcript models (Steijger et al. 2013), hampering evolutionary conservation analysis, transcript quantification, and differential expression analyses. Additionally, lncRNAs typically exhibit highly tissue- or condition-specific expression (Cabili et al. 2011; Derrien et al. 2012). As a consequence, the vast majority of lncRNAs can remain undetected if samples are not enriched for specific cell types. Hence, despite sequencing enabling identification of poorly expressed genes, the extent and complexity of higher eukaryotic transcription remains elusive. Our motivation is to reliably uncover the fraction of the genome that is expressed, the complexity of this expression, and to better utilize this information for lncRNA annotation (Mattick and Makunin 2006; Clark et al. 2011; Kellis et al. 2014).

Together with the identification of thousands of novel genes, sequencing has been readily utilized in the study of alternative splicing (Bryant et al. 2012; Merkin et al. 2012). Alternative splicing is a mechanism in which a single gene can produce different transcript isoforms by combining together different exons. This is an essential regulatory process. Many splicing factors display embryonic lethality when knocked out, and genomic aberrations that alter splicing are associated with a plethora of diseases (Tazi et al. 2009). In transcript model reconstructions, e.g., using Cufflinks, Trinity, or StringTie (Trapnell et al. 2010; Grabherr et al. 2011; Pertea et al. 2015), isoform reconstruction is driven by sequence reads that cross exon–exon splicing junctions. Recent work (Pervouchine et al. 2015) suggests that when analyzing Mouse ENCODE RNA-seq data, it is possible to identify up to 200,000 mouse splice junctions unreported by GENCODE. This discrepancy demonstrates that many exons and splice isoforms are missing from current annotations and suggests further transcriptome complexity remains to be detected. If a splice junction is rare, then the union of two exons is likely to be poorly supported. In this scenario, most assemblers fail to correctly call the junction, leaving two exons as independent units. This problem is compounded by the fact that monoexonic transcripts are then often discarded (Denoeud et al. 2008; Cabili et al. 2011) by de novo transcript detection.

Many of these issues can be addressed using targeted RNA sequencing (CaptureSeq). CaptureSeq utilizes magnetic bead-linked oligonucleotide probes to dramatically enhance the abundance of selected transcripts. Targeted cDNAs hybridize to the probes facilitating the purification of the RNA of interest prior to conventional RNA sequencing (Mercer et al. 2011). Previously, we used the CaptureSeq strategy to enrich and sequence targeted fractions of the human transcriptome (Clark et al. 2015). This allowed the discovery of novel transcripts, expressed beneath coverage restrictions usually imposed by RNA-seq and the joining of previously fragmented annotations. In human leukemia cell lines (K562), 42.1% of the transcriptome was expressed below 0.0366 attomole/µL and was better quantified by CaptureSeq (Clark et al. 2015). This fraction of the transcriptome was enriched in cancer and other disease associated loci (Clark et al. 2015). Given the advantages of CaptureSeq to characterize and quantitate poorly annotated, lowly expressed and high tissue-specific human lncRNAs (Mercer et al. 2011; Clark et al. 2015), we applied this method to the much less annotated mouse transcriptome.

We prepared a CaptureSeq design with 190,689 probes comprising 28,228 known and predicted mouse lncRNAs annotations. Additionally, we created a second CaptureSeq design with 154,281 probes designed to target annotated splice junctions. We used these two probe designs to profile transcriptome complexity in five mouse tissues (including three brain subregions), revealing both novel lncRNA and coding transcripts and simultaneously correcting previous annotations.

## Results

The two CaptureSeq probe designs were used to perform targeted sequencing of the splice junctions and lncRNA loci of the mouse genome. The lncRNA design was generated considering a combination of annotated and predicted loci (Methods). This data set spanned 28,228 transcripts, an increase of ∼2.8-fold with respect to lncRNAs annotated in GENCODE (M4). Transcript coordinates and an accompanying annotation table of targeted lncRNAs are provided (Supplemental Data S1, S2, respectively). We used probes designed to perform targeted sequencing in five mouse tissues: brain, heart, kidney, liver, and testis. Typically lncRNAs are highly tissue specific and enriched in brain and testes (Cabili et al. 2011). Furthermore, distinct neuronal populations have characteristic lncRNA expression landscapes (Kadakkuzha et al. 2015). To better represent the expression extent of brain lncRNAs, we included in our experimental design three additional brain subregions: cerebellum, cortex, and olfactory bulb. Starting from a total of 576,916,753 read pairs, 330,138,368 were successfully aligned to the mouse genome. Principal-component analysis (PCA) and clustering analyses based on expression levels and correlation correctly recapitulated the expected sample-to-sample relationships (Supplemental Figs S1, S2). In samples in which we performed targeted sequencing of splice sites, we measured a three- to sixfold increase in reads crossing splice junctions as compared to the other samples (from ∼10% to ∼60% junction spanning reads) (Supplemental Fig. S3A). We measured a similar enrichment when compared to standard RNA-seq from Mouse ENCODE (Supplemental Fig. S3B).

The mapped reads together with GENCODE (M4) annotations were used to guide assembly of the transcriptome. Annotations of both lncRNA and splice junction CaptureSeq experiments were merged together to generate a comprehensive assembly of 59,206 genes encoding 137,562 transcripts. The resulting data set incorporated the full GENCODE (M4) annotations together with the genes defined in the CaptureSeq experiments. As the CaptureSeq design included monoexonic ESTs and cDNAs (Methods) derived from sense intronic regions of the genome, this comprehensive assembly includes a higher fraction of unspliced transcripts with respect to GENCODE (24.4% versus 17.0%). When considering the spliced component only, the average number of isoforms per gene was comparable with GENCODE annotations (3.8 versus 3.2, respectively). However, the average number of exons per transcript was higher than in GENCODE annotations (9.8 versus 7.5, respectively; Wilcoxon *P*-value <2.2 × 10^−16^) (Supplemental Fig. S4). This indicates that newly discovered exons are predominantly assigned to already characterized genes and do not constitute independent monoexonic isoforms. The average transcript length increased from 1909 nt in GENCODE to 2759 nt, in agreement with the increased exons detected (Supplemental Fig. S4). The phastCons (Siepel 2005) conservation scores decreased from an average of 258.7 in GENCODE to 234.9 (Wilcoxon *P*-value <2.2 × 10^−16^), suggesting that many of the newly discovered isoforms are likely to be recently evolved or less constrained than annotated genes. This is in agreement with previous reports that low abundance genes tend to have less evolutionary constraint (Subramanian and Kumar 2004; Gout et al. 2010). This comprehensive unfiltered transcriptome assembly is freely available to the community as Supplemental Data S3.

**Figure 1.**
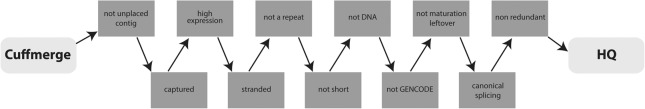
Filtering pipeline flowchart. The input is the comprehensive annotation returned by Cuffmerge. Then, we apply the series of 11 filters described in the text. The output is the high quality set (HQ).

We next sought to curate a high quality selection of novel transcripts (HQ set) by applying consecutive filtering steps to remove any mapping and assembly artifacts and to exclude previously annotated isoforms (Fig. 1).

Initially, we excluded transcripts mapping to unplaced mouse contigs (Methods). Then, we selected only those transcripts whose exons overlap at least one CaptureSeq probe (either in the lncRNA or splice junction design). Transcripts overlapping solely to control probes were discarded. Next, we discarded any poorly supported transcript isoforms. We utilized the known quantities of RNA spiked-in controls to set a threshold below which we consider the expression readings and transcript assemblies likely unreliable. The spike-in controls used were External RNA Controls Consortium (ERCC) RNAs (Baker et al. 2005). These consist of a set of unlabeled, polyadenylated transcripts of differing sizes and concentrations in order to measure against defined performance criteria. These controls have become widely accepted for normalization and quantitation of RNA samples.

Previous reports demonstrated that transcript models with at least eightfold sequence coverage can be confidently assembled (Jiang et al. 2011). Thus, for each sample, we estimated the known concentration and the FPKM for which the ERCC controls reach a lower limit of 8× sequence coverage (Supplemental Figs. S5, S6). Transcripts consistently expressed below the established sample-specific FPKM thresholds were discarded. We next removed all transcripts for which Cufflinks/Cuffmerge failed to predict the transcript orientation. This is mostly the case for monoexonic and/or weakly supported transcripts. We then filtered out transcripts located in highly repetitive or low complexity areas (Methods). Although the vast majority of the transcripts had a low content of repeats and low complexity regions, we nevertheless discarded all transcripts that included >90% masked nucleotides (Supplemental Fig. S7B). As the RNA-seq library preparation protocol does not reliably measure small RNA species (Ozsolak and Milos 2011), we removed all isoforms shorter than 200 nt (Supplemental Fig. S7A).

DNA contamination is an important consideration when detecting transcripts from sequencing data. Even with DNase treatment, it is possible for such contamination to produce short spurious monoexonic transcripts. Hence, we applied a filter to detect the presence of DNA contamination artifacts in our data. We discarded monoexonic intergenic transcripts without at least 80% of their reads mapping on the annotated strand in at least one sample. This filter affected only 45 transcripts, suggesting that most monoexonic transcripts in the set are not in fact DNA contamination (Supplemental Fig. S8). We then removed transcripts already annotated in GENCODE (M4) (Methods). Similarly, we tried to prevent the inclusion in the data set of maturation leftovers of previous genes. We discarded the monoexonic transcripts embedded into introns and expressed in the same orientation of the host genes without independent supporting evidence for transcriptional initiation (either overlap with FANTOM Cap analysis of gene expression [CAGE] tags) (The FANTOM Consortium and the RIKEN PMI and CLST [DGT] 2014) or expressed enhancers (Methods; Villar et al. 2015).

Next, we used gffread tool of the Cufflinks package to verify annotations and discard multiexon transcripts that have any intron with a noncanonical splice site (i.e., not GT-AG, GC-AG, or AT-AC). Finally, we collapsed highly redundant transcript isoforms differing just by a few nucleotides (Methods). A summary of the number of genes and transcripts discarded and retained at each filtering step is shown (Table 1).

**Table 1.**
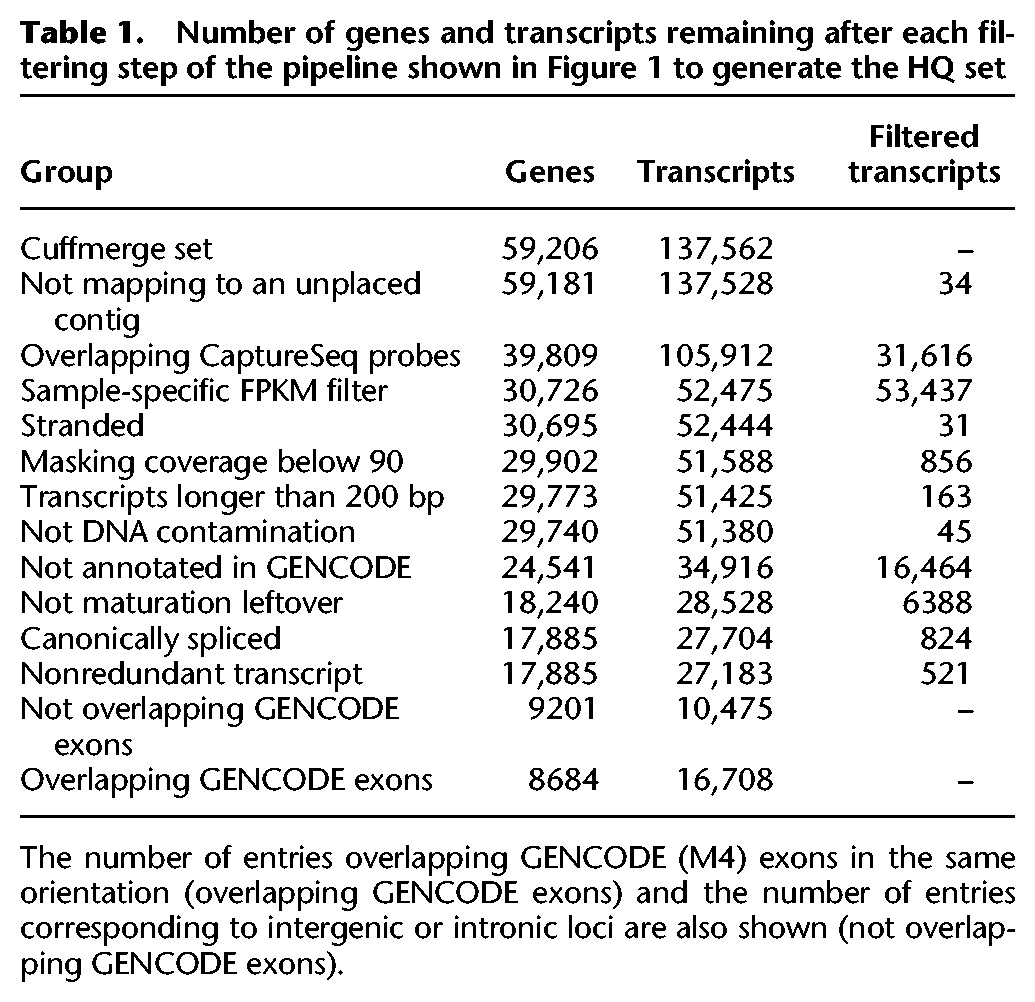
Number of genes and transcripts remaining after each filtering step of the pipeline shown in Figure 1 to generate the HQ set

The final HQ assembly comprises 17,885 genes and 27,183 novel transcripts not present in GENCODE. These annotations include both novel transcripts from known GENCODE genes and novel loci (Supplemental Fig. S9). In this set, 18,597 transcripts are spliced, whereas the remaining 8586 are expressed as single exons. This filtered set of high quality novel transcripts is provided (Supplemental Data S4) together with relevant genomic and expression features (Supplemental Data S5). Next, we sought to establish any protein-coding potential of the HQ transcript set utilizing the CPAT algorithm for the detection of coding potential (Supplemental Fig. S10A; Wang et al. 2013). These results suggest that 11,756 transcripts have at least some significant coding potential, and 15,427 are true lncRNAs. This prediction is also supported by BLASTx (Altschul et al. 1990) and rpstBlastN (Marchler-Bauer et al. 2002) analyses (Supplemental Fig. S10B). The coordinates of coding and noncoding transcripts, respectively, are provided (Supplemental Data S6, S7).

**Figure 2.**
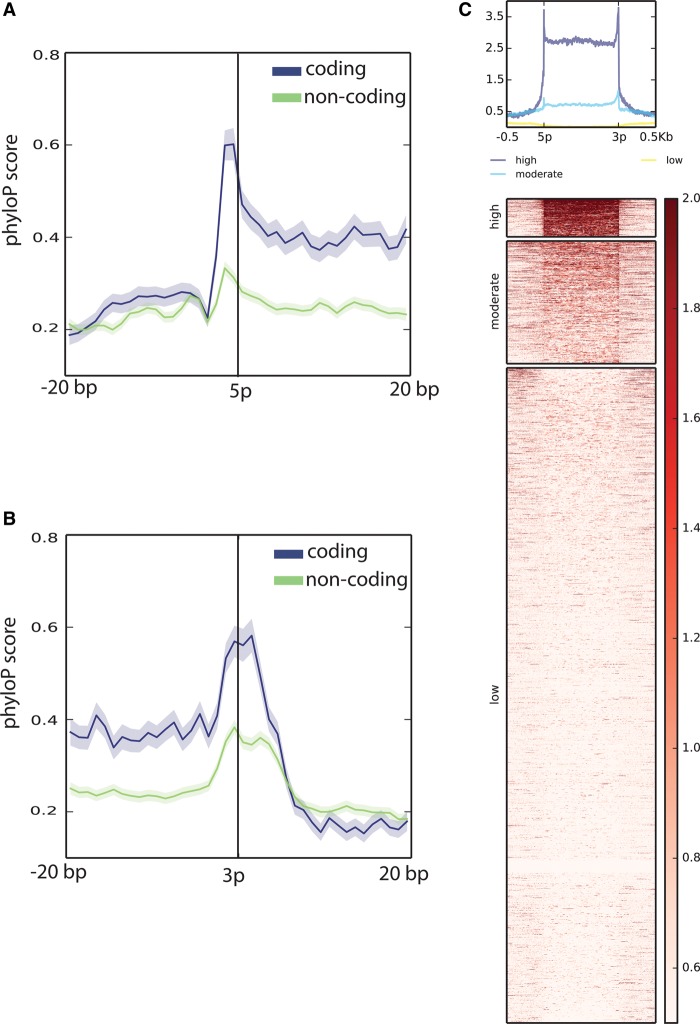
Evolutionary conservation of HQ spliced exons not overlapping previous exon annotations on either strand. 5′ (*A*) and 3′ (*B*) ends of the exons. The vertical axis shows the mean phyloP conservation score (Pollard et al. 2010), which measures the conservation of each single nucleotide independently of its context. The shading indicates the standard error. Coding exons are shown in blue. The expected ORF triplet pattern (Clark et al. 2015) is noticeable as a series of conservation peaks. Noncoding exons are shown in green. For both coding and noncoding exons, conservation spikes are visible on the two first nucleotides outside the exons, suggesting the presence of conserved splice donor/acceptors. Exons are labeled as coding if the CPAT predicted ORF coverage is >70%. Exons are labeled as noncoding if the CPAT predicted ORF coverage is <20%. Exons with ORF coverage between 20% and 70% are not shown in *A* and *B*. In *C*, each row of the heatmap represents an exon plus 500 bp upstream of and downstream from the 5′ and the 3′ ends. Each exon is scaled to fit to a region of 1000 bp. Each row is divided into bins of 1 bp. The color of each bin reflects the mean phyloP vertebrate conservation. The heatmap shows a scale of colors saturating below 0.5 and above 2. Missing data are set to a score of 0. The three divisions of the heatmap reflect the *k*-means clustering. The rows are sorted in descending order considering the mean value in each row. The bar on the *right* of the heatmap indicates the scale range. The profile on *top* recapitulates the mean conservation score at the level of each bin.

The population of HQ exons originating from spliced transcripts and not overlapping GENCODE (M4) exon annotations in any strand comprises both coding and noncoding elements (Methods; Fig. 2A,B; Supplemental Fig. S11). These exons display three distinctive conservation patterns (Fig. 2C). The first group (comprising 657 exons) shows marked evolutionary conservation compared with their surrounding genomic context and a median ORF coverage 38.2% (Methods). The second group includes 1974 exons with moderate conservation and lower ORF content (median coverage 24.1%). Interestingly, the last group includes 10,259 exons overlapping mouse-specific regions and repeats (Supplemental Fig. S12). This group is likely to be enriched in nonfunctional elements or RNAs for which their sequence is not necessary to exert their function. Interestingly, we also observed a conservation of the splice sites for the exons of the first two groups, consistent with what was previously described (Nitsche et al. 2015).

The HQ set was then divided into two groups to separate transcripts not overlapping any GENCODE exons from those representing alternative isoforms of known genes. Our capture arrays were designed against known or predicted gene loci, and so we would not expect to identify a large number of novel loci. Nevertheless, we can confirm the expression of 9201 loci previously unreported in GENCODE (M4), mostly corresponding to intergenic (53.9%) and intronic (24.8%) regions. In total, 1339 intergenic loci are also absent from additional comparison data sets (Supplemental Table S1). Similarly to what was already shown (Cabili et al. 2011; Derrien et al. 2012; Clark et al. 2015), the set of spliced intergenic noncoding RNAs (61 genes, 70 transcripts not found in GENCODE or the other catalogs) have a pronounced tissue specificity expression pattern (Supplemental Fig. S13). This fraction of intronic transcripts is mainly caused by probes designed to target GenBank (Benson et al. 2014) mRNA regions (Methods).

**Figure 3.**
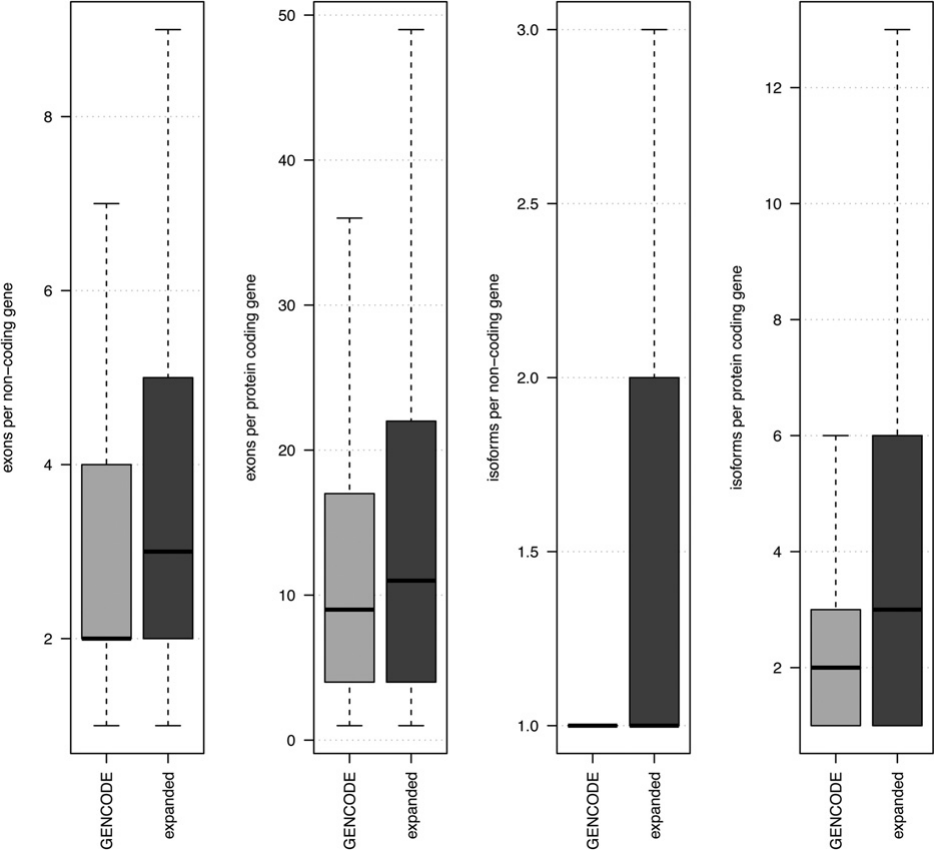
GENCODE annotation expansion. The light gray box plots indicate GENCODE (M4) annotations. The dark gray box plots indicate GENCODE genes combined with HQ genes. From *left* to *right*, the first panel shows the number of exons per annotated GENCODE noncoding gene. The second panel shows the number of exons per annotated GENCODE protein-coding gene. The third panel shows the number of transcript isoforms per annotated GENCODE noncoding gene. The fourth panel shows the number of transcript isoforms per annotated GENCODE protein-coding gene. All the comparisons are statistically significant with a Wilcoxon *P*-value <0.05.

We found 8684 genes (16,708 transcripts) overlapping known GENCODE exons on the same strand, comprising refined and expanded annotations achieved using CaptureSeq. These include totally novel unreported exons (8807), different isoforms of known exons (8981) and novel splice junctions (11,933). The compendium originating from the combination of these 16,708 transcripts and GENCODE (M4) is significantly more complex than GENCODE alone (Fig. 3; Supplemental Fig. S14). Overall both coding and noncoding genes show an increased number of exons or isoforms per gene (Fig. 3).

**Figure 4.**
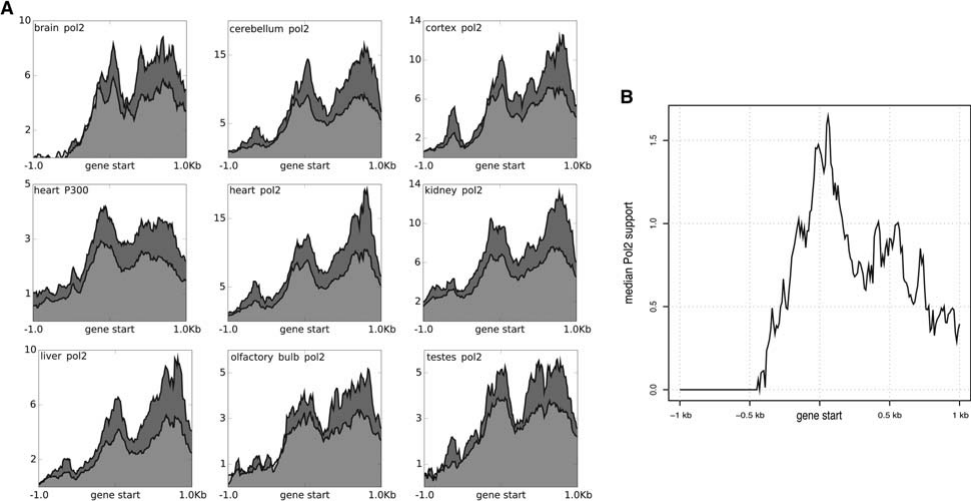
ChIP-seq support of the new gene starts. Same layout as in the profile of Figure 2C, but in this case, the bins are of 10 bp, the plots are centered on the gene starts, and they show a surrounding area of ±1 kb. (*A*) The light gray curve represents the mean ChIP-seq support across all the HQ genes. The dark gray curve represents the mean ChIP-seq support across just the HQ genes expressed in that specific tissue (FPKM >5). (*B*) The curve shows the median ChIP-seq support of the HQ genes in the tissue where they are the most highly expressed.

In this context, some of the HQ isoforms extend GENCODE genes by adding alternative starts and ends. In total, 816 HQ transcripts overlap one-to-one with GENCODE genes and their expression begins at least 400 nt upstream of the annotated start (Methods). To independently validate alternative transcription start sites, we analyzed Mouse ENCODE ChIP-seq profiles and DNase I hypersensitivity (DHS) data on tissue samples matching those considered in this work. A total of nine RNA polymerase II and EP300 ChIP-seq data sets (Fig. 4A) along with five DHS data sets (Supplemental Fig. S15A) were used (Methods). In all samples considered, the results show a correspondence between new gene starts and read density peaks. Since the HQ gene set resulted from a combination of transcripts expressed in different tissues, some of the HQ transcripts will not be expressed in each tissue utilized for validation. As expected, when each data set was reanalyzed with just the transcripts expressed in that tissue, the peaks became more pronounced (Fig. 4A; Supplemental Fig. S15A). Similarly, we repeated the analysis by selecting the tissue in which each gene reached its maximal expression level and then asked whether the gene has ChIP-seq/DHS support in that tissue. The results show a clearer overall picture, with a marked peak corresponding to the novel transcriptional start sites (Fig. 4B; Supplemental Fig. S15B). As a complementary approach, we used FANTOM cage peaks to validate new TSSs. For this analysis, we considered a set of 16,752 nonredundant transcript start sites mapping at least 400 nt away from annotated GENCODE transcript starts. In total, 4087 TSSs from this set are supported by at least one CAGE peak within 200 nt (Methods). We performed 1000 randomizations of the TSS coordinates to the mouse genome to test the null hypothesis that the CAGE peaks overlap TSSs by chance. This result is highly significant (empirical *P*-value <0.001) (Methods; Supplemental Fig. S16).

The results also support the presence of 1080 HQ genes (matching the same number of GENCODE genes), terminating at least 400 nt downstream from the annotated end. Interestingly, for some cases, this increased sequence coverage of lncRNAs and known junctions allowed bridging between neighboring genes, previously thought to be independent units. We provide the 400 HQ genes that correspond to two or more neighboring GENCODE genes in the same orientation (Supplemental Data S8). In 180 cases, the HQ genes merged together adjoining protein-coding genes, and in 50 cases, the merged genes belong to other GENCODE types, including lncRNAs and transcribed processed pseudogenes. One hundred seventy HQ genes merge together combinations of coding and other GENCODE types. One remarkable example is the bridging of *Grk4* to *Htt*, (G protein-coupled receptor kinase 4 and huntingtin) (Supplemental Fig. S14B). The splice junction connecting the two genes is supported by 14 and five reads, respectively, in olfactory bulb and cortex (Supplemental Fig. S17). Although interesting, it would be important for this finding to be validated with an orthogonal approach, e.g., PCR or 5′ RACE.

The subset of 3253 HQ genes enriched in brain is associated to known protein-coding genes involved in the functioning and development of the nervous system (Methods; Supplemental Fig. S18). For instance, *TCONS_ 00132850* and *TCONS_00132849* are brain-specific HQ transcripts divergently expressed from *Tspan7*. The gene *Tspan7* encodes for Tetraspanin 7, a transmembrane protein associated to X-linked mental retardation (Supplemental Fig. S19A; Holinski-Feder et al. 1999; Zemni et al. 2000; Abidi et al. 2002; Maranduba et al. 2004). Another example is represented by *TCONS_00134637* and *TCONS_00134636*, a brain-specific HQ transcript expressed on the promoter of the protocadherin gene *Pcdh11x* in antisense orientation. *Pcdh11x* is crucial for the activity of the central nervous system, and its mutation is linked to female epilepsy and cognitive impairment (Supplemental Fig. S19B; Dibbens et al. 2008).

**Figure 5.**
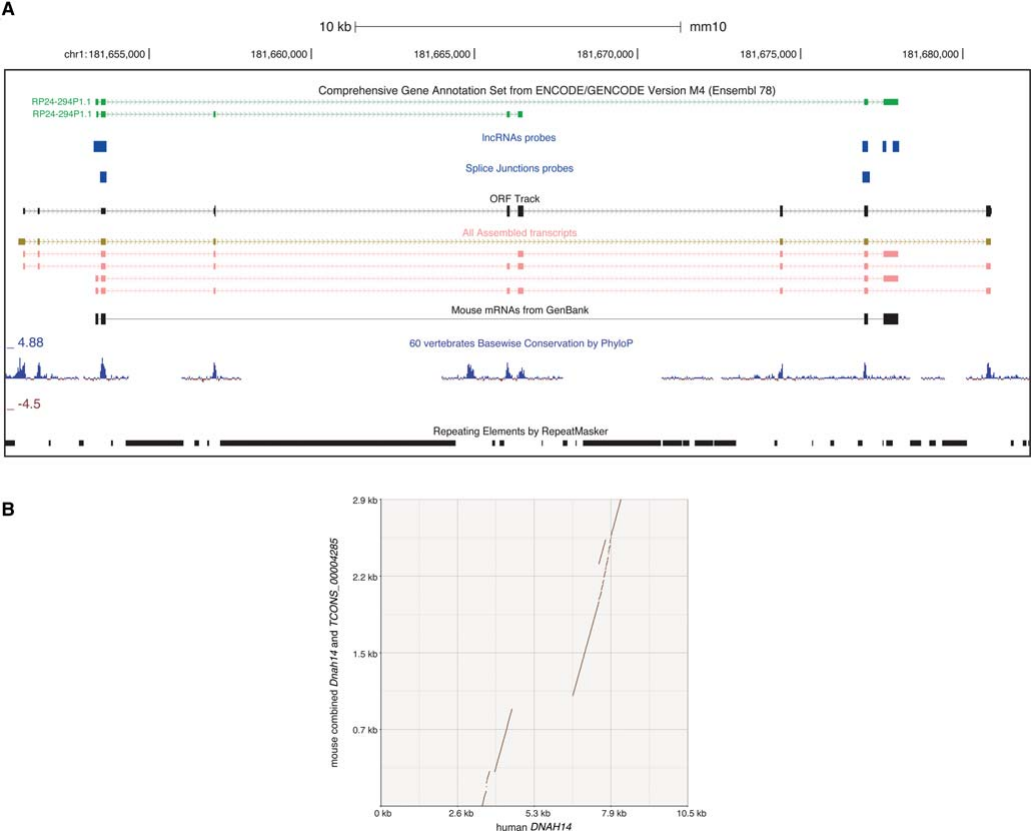
UCSC Genome Browser (Kent et al. 2002) snapshot. (*A*) Example of a GENCODE lncRNA, whose matching HQ gene shows protein-coding capability. From *top* to the *bottom*: the GENCODE (M4) annotations, lncRNA and splice junction CaptureSeq probes, the predicted ORF, all the assembled transcripts, GenBank mRNAs, phyloP conservation, and RepeatMasker (Smit et al. 2013–2015) tracks. The “ORF Track” reports the longest ORF predicted with Pinstripe (Gascoigne et al. 2012). “All assembled transcripts” highlights in gold the HQ transcript (*TCONS_00004285*) that adds new exons to the known lncRNA. (*B*) Dot plot alignment of the human *DNAH14* gene (*x*-axis) with a combination of mouse *TCONS_00004285* and *Dnah14*.

The expanded HQ annotations led to an increase in the ORF sizes of 594 known lncRNAs, allowing CPAT to reclassify a number as protein coding. Thirty GENCODE lncRNAs without any substantial coding potential are now predicted to form 30 protein-coding transcripts in the HQ transcript set (Fig. 5; Supplemental Data S9). Similarly, the inclusion of exons can result in the disruption of previous ORFs, thus supporting the lack of protein-coding capability of the annotated lncRNA (965 cases) (Supplemental Fig. S20). A summary of the relevant statistics of genes and transcripts in the HQ set is shown (Supplemental Table S2).

The overall conservation (phastCons) of the refined assembly is lower than when compared to previous annotations (Wilcoxon *P*-value 9.7 × 10^−134^) (Supplemental Fig. S21). This suggests that with CaptureSeq, we have succeeded in identifying weakly expressed or fast evolving isoforms of GENCODE genes otherwise undetected with standard RNA-seq and conservation approaches. Similarly, the nucleotide sequence of the HQ intergenic monoexonic transcripts shows low levels of evolutionary conservation (Supplemental Fig. S22).

## Discussion

In this work, we generated and analyzed high coverage RNA sequencing of mouse lncRNAs and splice junctions via targeted RNA sequencing. This approach has significantly improved on previous gene annotations and further highlights the extraordinary complexity of the transcriptional landscape in mouse.

We generated a comprehensive mouse lncRNA resource by merging current annotations, homology predictions, and RNAs lacking coding potential from various sources. We designed probes to target these lncRNA loci, trying to address the common problem of poor sequencing coverage that can hamper reliable detection of low abundance transcripts. Additionally, we designed probes to cover annotated splice junctions with the aim of expanding and improving the catalog of alternative splice sites for mouse. To this end, we sequenced 16 RNA samples from five mouse tissues and three brain subregions with external RNA spike controls. These data together with GENCODE (M4) annotations were used to generate a novel and comprehensive transcriptome assembly.

The linear relationship between known concentrations of RNA controls and measured quantities suggests that we have reliably measured transcripts whose expression lies in a range between 0.06 and 117.19 attomole/µL. This remarkable sensitivity is due to the use of CaptureSeq technology and has expanded considerably the number of exons and isoforms associated to known genes (Fig. 3). Additionally, we describe a comprehensive computational pipeline, containing extra safeguards and filtering steps. This system can detect previously unannotated transcripts and reduce their number to a small high quality set suitable for further validation. Given the many artifacts still present in modern sequencing and transcriptome assembly approaches, conservative filtering is extremely important.

In the set of high-confidence transcripts, 20.4% of the newly identified exons show evidence of negative selection and are more highly conserved than the genomic context (clusters high and moderate of Fig. 2C). This selective pressure is noticeable for exons at 5′/3′ transcript extremities, suggesting conservation of the splice acceptor and donor functions. As expected, new exons containing ORFs tend to exhibit stronger purifying selection (Supplemental Fig. S11). Surprisingly, the newly detected intergenic monoexonic transcripts are not as conserved as these novel exons (Supplemental Fig. S22). Given their intergenic location, it is unlikely these transcripts are degradation products of unknown genes. The filtering pipeline also ruled out the possibility that the monoexonic intergenic transcripts in the high-quality set represent DNA contamination, low complexity/repeat artifacts, or short spurious read mappings. One possibility is that elements in this set represent species-specific lncRNA or RNAs whose sequence is not required for their function.

With CaptureSeq, we succeeded in the detection of transcripts with concentrations as low as 0.02 attomole/µL (Supplemental Fig. S5); deeper sequencing would still be needed to fully capture the lowest end of the transcriptome expression range. For example, the HQ transcript *TCONS_00004285* improves on the annotation of *Gm42415* by adding novel conserved exons (Fig. 5A). Yet this annotation is likely to be incomplete. The adjacent gene is the heavy chain 14 of dynein (*Dnah14*, 17 exons), which is annotated in GENCODE as representing just a fragment of a bigger dynein sequence for which there is insufficient evidence. We compared the much longer human *DNAH14* homolog (136 exons) with a combination of mouse *TCONS_00004285* and *Dnah14* (Fig. 5B). On one hand, this analysis shows that *TCONS_00004285* aligns with the human dynein and constitutes an additional fragment of *Dnah14*. On the other hand, there is still a gap between the two parts, suggesting that some exons are still missing.

Although the CaptureSeq methodology represents a new paradigm for transcript annotation and detection, many challenges still lie ahead. In particular, the problem of RNAs expressed in very specific cell types or developmental stages remain difficult to quantitate and characterize. However, we propose that other model organism annotations could benefit greatly by the CaptureSeq strategy as described herein and as shown previously for human transcripts (Clark et al. 2015). These novel transcripts together with revised and improved annotations can shed more light onto the multifaceted transcriptional landscape of the murine genome and our understanding of transcriptional regulation in general. For example, 1193 of these high-quality novel transcript models are associated with Ensembl genes linked to disease (Yates et al. 2016). We believe the methods, approaches, data, and annotations generated by this study will be of significant benefit to the mouse community.

## Methods

### LncRNA Capture design

Oligonucleotide-probe target regions were designed to capture all mouse lncRNAs. Mouse lncRNAs were identified by updating and extending the method outlined in Clark et al. (2012). Mouse RNAs were obtained from the following sources: the UCSC mm9 mRNA track (alignments between GenBank RNAs and the genome), UCSC genes (predictions based on RefSeq, GenBank, and the tRNA Genes track) (Kent et al. 2002), and RefSeq genes (Pruitt et al. 2012) (downloaded April 10, 2012); also included were lincRNAs identified in Belgard et al. (2011), transcripts overlapping novel lincRNA loci identified in Guttman et al. (2010), and transcripts overlapping lincRNA loci investigated by Guttman et al. (2011).

In the initial classification, transcripts with <5% overlap with protein-coding exons from RefSeq and UCSC genes and an ORF less than 100 amino acids were putatively defined as noncoding. Any transcripts of known lncRNAs in lncRNAdb (Amaral et al. 2011) were classified as noncoding. All transcripts >200 nt were selected for further filtering. Further filtering steps involved the following:

1. Any transcripts with >95% antisense overlap to a RefSeq coding gene were removed (removes many “mirror” transcripts and 3′ UTR transcripts with an incorrectly determined strand).
2. All multimapping or split chromosome mapping RNAs were removed.
3. Cuffcompare (Trapnell et al. 2010) was utilized to create a nonredundant transcript set. Any transcripts classified as “s” or “p” (if they were downstream from a RefSeq coding gene) were removed.
4. The coding potential programs CPAT (Wang et al. 2013), *CPC* (Kong et al. 2007), and PhyloCSF (Lin et al. 2011) were run to classify transcripts as coding or noncoding. Using cutoffs of CPAT: 0.44, CPC: 0, and PhyloCSF: 20, any transcript called coding by two or more methods was removed. Since not all transcripts have valid *CPC* and PhyloCSF scores, any transcript with a CPAT score only, and which was above the cutoff, was also removed.
5. The most highly expressed putative lncRNAs were manually examined, and any which appeared to be 3′ UTRs were removed.
6. Any transcripts present in lncRNAdb that were removed in Steps 1–5 were reinstated.

In total 28,312 transcripts from 23,306 loci were classified as lncRNAs and targeted by the CaptureSeq array (Supplemental Data S1, S2, respectively). Of these, only 28,228 transcripts were successfully converted to the GRCm38 primary assembly using liftOver (Kuhn et al. 2013) and were used for all analysis. As part of the array design, monoexonic lncRNA regions overlapping or within 50 nt of a coding exon in the same orientation were trimmed to reduce off-target capture of coding genes. A number of controls were added to the array, including 1278 sequences targeting random lncRNA intronic regions (filtered to ensure targeted introns were >300 nt in length, target sequences did not overlap any known exons and were not >50% repetitive); a 100-kb gene desert region; 2000 promoter controls (500-nt randomly picked regions located 0.5–1 kb upstream of lncRNA start sites with no exonic overlap and which were not >50% repetitive); a 100-kb region of the *Escherichia coli* K12 genomic sequence; and the ERCC RNA Spike-In Control set (Life Technologies). Also present on the array were 6,438 putative mouse lncRNA regions with homology to human lncRNAs identified as previously described (Derrien et al. 2012) and not overlapping known RefSeq or Ensembl V64 coding exons. In total, the design targeted ∼53.1 Mb of the mouse MGSCv37 genome.

Probe selection and synthesis was performed by Roche/Nimblegen and allowed a maximum of five matches to the genome. The oligonucleotide capture probes covered 85.7% of target regions directly, with an estimated 91.3% of target regions able to be captured. Capture design probe coordinates were converted from MGSCv37 to GRCm38 assembly using liftOver and provided in Supplemental Data S10.

### Splice junction Capture design

The splice junction design aimed to capture sequence reads that traversed splice junctions. We initially retrieved all mouse gene annotations from the mouse genome (RefSeq genes, UCSC Genome Browser, mm9). We then retrieved coordinates for the 100-nt region upstream of the annotated 5′ splice site, or 100 nt downstream from the 3′ splice site. We then estimated the relative abundance of transcription within these regions using signal tracks (.wig) retrieved from the ENCODE/LICR RNA-seq data sets (Cerebellum, Cortex, Whole Brain). We ranked regions according to estimated abundance and removed the most abundant 15%. This step was performed to improve the enrichment potential of the design. Regions passing this step were considered target regions for the array, and overlapping target regions (from exons shorter than 200 nt) were merged. Highly repetitive sequences were removed from the final design according to NimbleDesign Sequence Capture Developer Guidelines (http://www.nimblegen.com/products/lit/06465528001_NG_SeqCap_Developer_Guidelines_v1p0.pdf). The genome coordinates of the probes were passed to GRCm38 assembly using liftOver (Kuhn et al. 2013). In total, probes overlapped the splice junctions in 19,201 genes, encompassing a total footprint of ∼26.5 Mb. The probe coordinates in GRCm38 are provided in Supplemental Data S11.

### Sample preparation and Capture sequencing

Mouse tissue samples were obtained from adult C57BL/6J mice (AEC Approval Number IMB/030/08/ARC). Mouse brain samples were dissected in ice cold PBS, snap frozen in liquid nitrogen, and RNA extracted with TRIzol (Life Technologies). Nonbrain organs were removed and placed in ice cold PBS, any contaminating tissue was removed, and the required organ cut into pieces <30 mg and placed in RNAlater (Qiagen). RNA was extracted using the TissueRuptor and RNeasy Mini purification kit (Qiagen). Purity of all mouse samples was validated by Nanodrop (Thermo Scientific). RNA was DNase treated with Turbo DNase (Life Technologies), purified, and confirmed DNA-free by PCR for gDNA as described in Mercer et al. (2014). Intact RNA was confirmed by Agilent 2100 Bioanalyzer (Agilent Technologies). Ribo-Zero (Epicentre) rRNA depletion was performed on 10 µg of each sample. For nonbrain samples, 5 µg of purified RNA from two organ pieces from the same mouse was ribodepleted and then pooled to ensure good coverage across the tissue. Efficient ribodepletion was confirmed by a Bioanalyzer (Agilent 2100) Pico chip.

Capture sequencing was performed as previously described (Mercer et al. 2015) combining the NimbleGen SeqCap EZ Library SR User's Guide V3.0 and the NimbleGen Arrays User's Guide: Sequence Capture Array Delivery v3.2 with the following specifications. RNA sequencing libraries were created using the TruSeq Stranded mRNA Sample Preparation Kit (Illumina). All samples contained ERCC spike-in mix no1 or no2 at a 1/100 dilution. Test libraries to estimate yield were amplified for 15 cycles as per “Enrich DNA fragments” protocol. Ten cycles of precapture LMPCR was performed for all samples except brain and liver (11 cycles). Capture hybridizations were performed as a four-plex with 250 ng of brain or nonbrain libraries pooled together. Post-LMPCR was performed for 17 (nonbrain samples) or 16 (brain samples) cycles, respectively. Quantitative PCR confirmed enrichment of target sequences and depletion of off-target sequences (Supplemental Fig. S23). The primer sequences are available in Supplemental Data S12. The libraries were sequenced on an Illumina HiSeq machine (2 × 102 bp paired-end reads) and produced on average 36,057 million pairs of reads (mean fragment length 144.066; mean standard deviation 37.29).

### Read mapping and transcript assembly

We used TopHat2, (version 2.0.13; --no-coverage-search; --b2-sensitive) (Kim et al. 2013) to align reads against the mouse genome (GRCm38) and a prebuilt transcriptome index generated using GENCODE annotations (M4, released December 3, 2014). We used Cufflinks (v2.2.1) to perform a Reference Annotation Based Transcript assembly (RABT) of reads mapped in each of the 16 CaptureSeq samples. We then merged the individual transcriptome assemblies into a comprehensive assembly using Cuffmerge (v2.2.1). The resulting assembly comprised 137,562 transcript and 59,206 gene models. We then filtered this assembly to discard transcripts on unplaced contigs. Although the unplaced contigs are valid sequences and the capture design includes probes mapping to them, we excluded these transcripts from the HQ set to allow the comparison with publicly available resources that do not make use of unplaced contigs.

### Sample-to-sample distance

Gene expression count quantitation was performed using HTSeq count (v0.6.1) (Anders et al. 2015) on genes across the 16 samples. These count data were normalized using DESeq2 (v1.6.3) (Love et al. 2014), and Euclidean distances between the samples were calculated on these regularized log-transformed counts. The distance matrix was used to compute sample-to-sample distances. The BioConductor package FactoMineR (v1.29) was used to perform principal component analysis on this matrix.

### Repeat and low complexity filtering

To generate the HQ set, we removed transcripts with a high content of repeats or low complexity elements. Such regions were defined according to RepeatMasker (Smit et al. 2013–2015) masked nucleotides of Ensembl version 74 assembly. Transcripts showing >90% masked nucleotides were removed.

### Filtering of previously annotated transcripts

To generate the HQ set, we removed transcripts already annotated in GENCODE (M4). To identify such transcripts, we utilized the BEDTools2 intersect (Quinlan and Hall 2010) (v2.22.1) tool with options “-s –split –f 0.99.”

### Filtering of redundant transcripts

To generate the HQ set, we removed redundant transcript isoforms. The Jaccard distance was used to assess isoform dissimilarity, comparing the union and the intersection of the transcript coordinates. For each gene, we removed all the isoforms with a Jaccard distance score >0.98 as defined by BEDTools2 (v2.22.1) (Quinlan and Hall 2010).

### FPKM threshold estimate

Transcript assemblies with less than eightfold coverage can be considered unreliable and discarded (Jiang et al. 2011). To estimate an FPKM threshold corresponding to eightfold coverage, we calculated a second-degree polynomial fit between mean ERCC coverage [log_2_(read counts × read length/ERCC size)] and known concentrations (log_2_) for each sample; based on this, we calculated the concentration for which the fit value equals 8 (Supplemental Fig. S4). We then computed a third-degree polynomial fit between known ERCC concentrations and their FPKM values calculated by Cufflinks; based on this fit, we calculated the fitted FPKM value at the concentration for which the coverage is eightfold (Supplemental Fig. S5).

The polynomial fits were calculated in R (version 3.1.2) using the lm (linear model) function. Based on the known concentrations of ERCC spike-ins, we are able to provide an approximate estimate of the RNA copy number at which eightfold coverage is reached. In liver, an eightfold coverage is reached at 0.02 amol/µL, which taking into account a total volume of 24 µL, corresponds to 2.89 × 10^5^ molecules (0.02 amol/µL × 24 µL = 0.48 amol = 0.48 × 10^−18^ mol; 0.48 × 10^−18^ mol × 6.022 × 10^23^ molecules/mol = 2.89 × 10^5^ molecules). Considering that the starting amount of RNA is 10 µg and assuming an RNA extraction yield for mouse liver of 5 µg/mg of tissue (Qiagen RNeasy Mini Handbook), we can estimate that the 10 µg of RNA used for library preparation were extracted from 2 mg of tissue (10 µg/5 µg/mg = 2 mg of liver tissue). Assuming an hepatocellularity number for mouse liver of 1.35 × 10^8^ cells/g tissue (Sohlenius-Sternbeck 2006), we can estimate that 2 mg of tissue correspond to 2.7 × 10^5^ liver cells (0.002 g × 1.35 × 10^8^ cells/g = 2.7 × 10^5^ cells). Therefore, it can also be estimated that 8× sequencing coverage is reached at a concentration of one copy of RNA per cell (2.89 × 10^5^ molecules/2.7 × 10^5^ cells = 1.07 molecules/cell).

### Splicing maturation degradation product filtering

To prevent the inclusion in the HQ set of possible degradation products of splicing events, we removed sense intronic monoexonic transcripts lacking 5′ CAGE support and with no evidence of being expressed enhancers. Transcripts whose transcription initiation did not match any permissive FANTOM CAGE peak (The FANTOM Consortium and the RIKEN PMI and CLST [DGT] 2014) within 100 bp distance and not sharing at least 80% reciprocal coverage with mouse enhancers (Villar et al. 2015) were discarded.

### Conservation analysis

The UCSC (Karolchik et al. 2004) *phastConsElements60way* track was used to estimate transcript evolutionary conservation (Supplemental Figs. S4, S20). This considers phastCons-conserved elements generated using multiple alignments of 59 vertebrate genomes to the mouse GRCm38 genome. For each transcript, the scores of the conserved elements are multiplied by the number of overlapping bases and added. The total score is normalized by the sum of the exon lengths.

The conservation analysis shown in Figure 2 was made with the set of HQ spliced exons not overlapping GENCODE (M4) exons in any strand. The analysis was performed with deepTools (v1.5.9.1) (Ramírez et al. 2014) and the 60-way phyloP (Pollard et al. 2010) conservation bigWig (mm10.60way.phyloP60way.bw) available from UCSC. deepTools was run with the option –binSize 1 to measure the conservation with 1 nucleotide resolution. To reduce the redundancy of the data set and avoid double counting, the coordinates of overlapping exons were collapsed with BEDTools2 merge (Quinlan and Hall 2010) (v2.22.1) to the leftmost and rightmost positions. The promoter conservation analysis shown in Supplemental Figure S25 was made with deepTools (v1.5.9.1) together with the phyloP 60-way conservation bigWig, 10-nt bin resolution and showing an interval of 1 kb (±500 bp around the TSSs).

### Coding potential analysis of HQ exons originating from spliced transcripts

The maximum ORF size of HQ exons derived from spliced transcripts was estimated with CPAT. In Figure 2A and B, exons are labeled as coding if the ORF covers at least 70% of their sequence. Exons are marked as noncoding if the ORF covers <20% of their sequence. ORFs covering between 20% and 70% of the exon sequences are not shown. Figure 2C shows three groups of exons based on evolutionary conservation. The medians of the ORF coverage of the exons in these groups were measured using CPAT maximum ORF size predictions.

### Selection on HQ genes starting upstream of the previous annotation

The selection of HQ genes corresponding to annotated genes but showing an upstream start consider multiple steps. First, the coordinates of GENCODE (M4) genes overlapping on the same strand were collapsed into single units. Next, the HQ genes overlapping one-to-one with either GENCODE (M4) genes or collapsed blocks and starting at least 400 nt upstream were selected. By doing the gene bridging, two or more separate GENCODE (M4) genes were discarded. Finally, the exon coordinates were compared to make sure that there is a match between HQ exons and previous exons.

### Mouse ENCODE data processing

We use the following RNA polymerase II ChIP-seq data sets downloaded from the Mouse ENCODE portal (http://hgdownload.cse.ucsc.edu/goldenPath/mm9/encodeDCC/wgEncodeLicrTfbs/): cerebellum, cortex, heart, kidney, liver, olfactory bulb, testis, and whole brain. This is combined with EP300 ChIP-seq from heart and the Input samples. DHS data sets were downloaded from the Mouse ENCODE portal (http://hgdownload.cse.ucsc.edu/goldenPath/mm9/encodeDCC/wgEncodeUwDnase): brain, cerebellum, heart, kidney, and liver. In both cases, reads were remapped to the GRCm38 assembly with Bowtie 2 (--very-sensitive option set) (Langmead et al. 2009). We aligned a total of 76 samples. Alignments from multiple samples corresponding to the same tissue were merged together and transformed to bigWig format. The ChIP-seq samples were scaled by library size and Input subtracted using bigwigCompare in the deepTools package (Ramírez et al. 2014).

Figure 4 demonstrates the ChIP-seq scaled and Input subtracted scores for the HQ genes starting upstream of the previous annotations. The panels in Figure 4A represent the mean ChIP-seq scores, in which each panel refers to a different tissue. The light gray slopes indicate all the HQ genes starting upstream of the previous annotations. The dark gray slopes represent tissue-specific subsets, including just the genes expressed with at least 5 FPKM in each specific tissue. In Figure 4B, the curve shows median score. In this case, each gene contributes just with the score of the tissue where it is best expressed (highest FPKM).

Similarly, Supplemental Figure S15 shows the DHS support of the HQ genes starting upstream of the previous annotations. In (a), each panel represents a different tissue. The purple slopes represent mean DHS values for all the HQ genes starting upstream of previous annotations. The orange curves represent tissue-specific subsets, including for each tissue just the genes expressed with at least five FPKMs. The curve shown in (b) represents the median DHS scores, in which each gene contributes with the DHS score of the tissue where it is best expressed (highest FPKM).

Two brain RNA-seq replicates (GEO accession GSM1000572) were downloaded from http://hgdownload.cse.ucsc.edu/goldenPath/mm9/encodeDCC/wgEncodeCshlLongRnaSeq, and the reads were aligned to the mouse GRCm38 genome using TopHat2, (version 2.0.13; --no-coverage-search; --b2-sensitive) in combination with a prebuilt transcriptome index generated using GENCODE annotations (M4, released December 3, 2014).

### LncRNA expression sensitivity analysis

In Supplemental Figure S24, the two brain RNA-seq libraries (GEO accession GSM1000572) were used to compare the lncRNA expression sensitivity. To define the lncRNA gene set, the HQ transcript models corresponding to GENCODE M4 lncRNAs were selected using BEDTools2 intersect (version 2.25.0) (Quinlan and Hall 2010) with options “-split -s -F 0.8” (requiring at least 80% overlap between the GENCODE lncRNA and the HQ model and the same strand). The genes of the selected transcripts were represented as dots in Supplemental Figure S24. The expression readout was estimated using HTSeq count (version 0.6.1) gene counts added with 1 pseudo count, normalized by the total reads mapped by TopHat2, and natural logarithm transformed.

### CAGE support analyses

Permissive FANTOM (The FANTOM Consortium and the RIKEN PMI and CLST [DGT] 2014) CAGE peaks were downloaded from http://fantom.gsc.riken.jp/5/datafiles/latest/extra/CAGE_peaks/. The genomic coordinates were recomputed for the GRCm38 assembly using liftOver (Kuhn et al. 2013). We assessed whether the observed overlap between new transcription sites and CAGE peaks would be achievable by chance. Hence, we select a nonredundant HQ transcription start site (TSS) set. If two or more transcript start sites map at least 50 nt from each other, only the isoform with the highest FPKM was considered. Additionally, we discarded all TSSs mapping on the same orientation and within 400 nt from GENCODE TSSs. We randomly projected the TSS coordinates to the mouse genome 1000 times utilizing BEDTools2 shuffle (Quinlan and Hall 2010) (v2.22.1), excluding the gap regions, the random chromosomes, the unplaced contigs, and the mitochondrial chromosome. The overlap between the CAGE peaks and the selected genomic areas was performed with BEDTools2 window (v2.22.1) reporting just the overlaps on the same strand and within an area of 200 nt.

### Selection of brain enriched/depleted HQ genes and GO analysis

In this analysis, we considered the HQ genes enriched in the brain tissues (brain, cortex, cerebellum, and olfactory bulb) versus the nonbrain ones (heart, liver, kidney, and testes). Expression was measured with Cuffquant/Cuffnorm (Trapnell et al. 2012). We selected the HQ genes with a log_2_ fold change brain/nonbrain of the median FPKM above 2 (Equation 1) as follows:

(1) $$\mathrm{lo}g_{2}\left(\frac{Mb}{Ma}\right)>2\mathrm{FPKM},$$

where *Mb* and *Ma* represent, respectively, the median FPKM in brain and nonbrain tissues. The HQ genes with median FPKM below 2 in both the brain and nonbrain tissues were not considered in this analysis. For each of the remaining HQ genes, we collected the identifiers of the overlapping RefSeq gene when available, or the closest RefSeq genes mapping on the 3′ and on the 5′. The list of RefSeq identifiers was converted to Entrez-ids and evaluated for GO biological process term enrichment with R 3.1.2 using the library GOstats 2.32 and the R Bioconductor genomewide mouse annotations from package org.Mm.eg.db (version 3.0.0) (Falcon and Gentleman 2007; R Core Team 2014). The results were sorted by *P*-value, and the top 20 terms are shown. Each term is shown in a color scale representing the Benjamini-Hochberg multiple testing adjusted *P*-value (Benjamini and Hochberg 1995).

## Data access

The RNA-seq data from this study have been submitted to the NCBI Gene Expression Omnibus (GEO; https://www.ncbi.nlm.nih.gov/geo/) under accession number GSE72311 and to the NCBI BioProject (https://www.ncbi.nlm.nih.gov/bioproject/) under accession number PRJNA293710.
